# Supplementary material for: The role of age and gender in the relationship between personality traits, quality of life, and decision-making about orthognathic surgery—A cross-sectional study
Source: PLoS One. 2026 Jan 16;21(1):e0340828. doi: 10.1371/journal.pone.0340828 (PMC12810834; doi:10.1371/journal.pone.0340828)
Supplement: Attachment — Submitted filename: response to reviewer Jeta Kelmendi.docx [file pone.0340828.s002.docx]

**Manuscript ID:** PONE-D-25-27428
**Title (revised):** The Role of Age and Gender in the Relationship between Personality Traits and Quality of Life, and Decision-Making About Orthognathic Surgery – A Cross-Sectional Study

Dear Editor and Reviewers,

We are grateful for the constructive and detailed feedback provided. Below, we present our point-by-point responses.

## ****Reviewer 1****

**General comment:** The study is interesting but requires substantial revision to meet PLOS ONE standards.
We thank the reviewer for the constructive feedback. We have thoroughly revised the manuscript, including title, abstract, introduction, methods, results, discussion, and conclusions, to address all points raised.

### ****Title****

**Reviewer comment:** “No capital letters, no question format, type of study must be provided.”
Revised to: “The Role of Age and Gender in the Relationship Between Personality Traits, Quality of Life, and Decision-Making About Orthognathic Surgery – A Cross-Sectional Study.”

### ****Abstract****

**Reviewer comment:** Expand abstract (max 300 words), provide exact results and P values, and avoid speculation.
Abstract has been expanded, exact p-values, effect sizes, and 95% CI for OR have been reported. Conclusions are now limited to the study aims, without speculation: "The relationship between personality traits, QoL, and the decision to undergo orthognathic surgery was more influenced by age than gender. OF and FE primarily influence decision, regardless of age or gender”.

### ****Introduction****

**Reviewer comment:** Section headings should follow journal style; provide a valid null hypothesis.
Headings revised according to journal style. A null hypothesis has been added:
“The null hypothesis proposed that age and gender do not play a significant role in this relationship.”

### ****Methods****

**Reviewer comment 1:** Justify sample size.
**It has been now clarified in M&M section: “Sample size determination** was based on an a power calculation using an online calculator https://www.danielsoper.com/statcalc. With α = 0.05, power = 0.80, and an expected large effect size (Cohen’s d = 0.8), the minimum sample required per group was n=21 and 26 for one- and two-tailed hypotheses. Groups were subjects who accepted vs. refused surgery, separately for two genders or two age groups, so in total 84-104 participants.”

**Reviewer comment 2:** Pre-registration is missing.
Acknowledged as a limitation in the Discussion: “(…) the study was not preregistered, which we recognize as a methodological limitation.”

**Reviewer comment 3:** Use of “Caucasian ethnicity.”
Revised to “participants of European origin,” justified by the availability of validated Croatian-language instruments; acknowledged as a limitation for generalizability.

**Reviewer comment 4:** Provide more details for instruments.
Expanded descriptions of OQLQ, BFI-44, FMPS, and RSES with psychometric properties and references has been added in M&M section: “Instruments

Orthognathic Quality of Life Questionnaire (OQLQ)

The validated Croatian version of the OQLQ was used to capture four dimensions: oral function (OF), facial aesthetics concern (FA), social aspects of deformity (SA), and awareness of dentofacial deformity (AW) by using 22 items [11]. Each item was scored on a 4-point Likert scale, with higher scores indicating greater impairment. Internal consistency of Croatian OQLQ ranged from α=0.80-0.92 [11].

Big Five Inventory (BFI)

Personality traits were measured using the validated Croatian 44-item BFI [12], which assessed five broad personality dimensions with two facets for each trait: extraversion (facets assertiveness and activity), agreeableness (altruism and compliance), conscientiousness (order and self-discipline), neuroticism (anxiety and depression), and openness to experience (esthetics and ideas) [12,13]. Each item was rated on a 5-point Likert scale (1 = strongly disagree to 5 = strongly agree). The BFI has demonstrated robust psychometric properties across multiple cultural contexts, including European populations, with subscale internal consistency ranging between α = 0.70 and 0.85 [12].

Frost Multidimensional Perfectionism Scale (FMPS).

Perfectionism was assessed using the 35-item Croatian version of the FMPS [14]. The scale evaluated six dimensions: personal standards, concern over mistakes, doubts about actions, parental expectations, parental criticism, and organization. In addition, global perfectionism was calculated as a summary score of all dimensions except organization. Items were rated on a 5-point Likert scale (1 = strongly disagree to 5 = strongly agree). Previous studies in Croatian and European samples reported Cronbach’s α values between 0.74 and 0.89 for subscales [14].

Rosenberg Self-Esteem Scale (RSES).

Global self-esteem was measured using the 10-item RSES, validated in Croatian population [15]. Each item was rated on a 4-point Likert scale (1 = strongly disagree to 4 = strongly agree), with higher scores reflecting greater self-esteem. The RSES has shown high internal reliability (α >0.85) [15].”

**Reviewer comment 5:** Clarify consent.
This text has been added: “For participants ≥18 years, written informed consent was obtained. For those <18 years, written informed consent from a parent or legal guardian, along with participant assent, was required. The study protocol was approved by the Ethics Committee of the University of Rijeka Faculty of Dental Medicine (Approval No. 2170-57-006-20-01) and the Clinical Hospital Center Rijeka (Approval No. 2170-29-02/1-20-2).”

### ****Results****

**Reviewer comment 1:** Report exact p-values, revise figures/tables.
All p-values are now reported exactly. New figures have been made - boxplots separately for age groups and genders for differences in personality characteristics and QoL between subjects who accepted and refused surgery.

**Reviewer comment 2:** Ensure self-explanatory tables.
A table with baseline characteristics (Table 1), including demographics, and psychometric scores by age and acceptance has been now added.

### ****Discussion****

**Reviewer comment 1:** Clarify null hypothesis.
It has been now clarified at the start of the Discussion: “The null hypothesis has been rejected. This study reveals that age, as a marker of psychological development, has a more substantial influence on the relationship between personality traits, QoL and decision to accept orthognathic surgery for dentofacial deformities than gender.”

**Reviewer comment 2:** Low number of participants.
Addressed in Limitations: “(…) the sample size was modest, particularly for male participants, limiting generalizability of gender-related findings.”.

**Reviewer comment 3:** Generalizability.
Limited generalizability acknowledged due to sample size and ethnicity restriction, as the study included only Croatian participants to match the validated language versions of the instruments: “

### ****Conclusions****

**Reviewer comment:** Avoid speculation, repeat aims only.
 Conclusions revised: concise, limited to aims, no speculation. This text is now after Discussion: “The relationship between personality traits, QoL, and the decision to undergo orthognathic surgery was more strongly influenced by age than by gender, with several distinctions. Adults tended to accept surgery more often, driven by higher perfectionism and lower self-esteem, whereas these traits were not decisive among adolescents. In males, heightened awareness of deformity served as a motivation, while in females, lower self-esteem was influential. OF and FE primarily influence decision, regardless of age or gender.”

Shorter version is in Abstract: “ The relationship between personality traits, QoL, and the decision to undergo orthognathic surgery was more influenced by age than gender. OF and FE primarily influence decision, regardless of age or gender.”

## ****Reviewer 2****

**Reviewer comment:** Interesting and clinically relevant study. Limitation: predominantly female sample, gender effect may be underestimated.
We agree. Limitations now highlight that the gender imbalance (68% female, 32% male) may have reduced the power to detect gender effects: “(…) the sample size was modest, particularly for male participants, limiting generalizability of gender-related findings.”.

## ****Reviewer 3****

**Reviewer comment 1:** Funding inconsistencies.
Sorry for the mistake. This is the final statement that has been added: “This work was supported by the University of Rijeka Grant (uniri-biomed-18-22). The funder had no role in study design, data collection, analysis, interpretation, or manuscript preparation.”

**Reviewer comment 2:** Define age groups.
Adolescents defined as ≤17, adults ≥18; group sizes reported (n=46 and n=62).with group sizes of 46 and 62, respectively.

**Reviewer comment 3:** Consent for minors.
This text has been added: “For participants ≥18 years, written informed consent was obtained. For those <18 years, written informed consent from a parent or legal guardian, along with participant assent, was required.”

**Reviewer comment 4:** Clarify exclusion criteria.
Replaced “mental disabilities” with “clinically significant neurodevelopmental or cognitive impairment precluding valid questionnaire completion.”

**Reviewer comment 5:** Ethnicity restriction.
Justified as related to validated Croatian instruments; acknowledged as a limitation.All participants were of Croatian ethnicity to ensure cultural and linguistic validity of the psychometric instruments used . This homogeneity allows for comparability but may limit generalizability to other populations

**Reviewer comment 6:** Missing data.
Data completeness was checked before analysis. Missing responses were minimal (<5% per instrument). Cases with missing data were excluded pairwise in relevant tests, without imputation. Data were screened for completeness and accuracy prior to analysis. Participants with missing responses in key instruments were excluded from specific analyses. The level of missing data was low and did not affect the validity of the results. It has now been clarified in the text.

**Reviewer comment 7:** Report effect sizes and CI
Effect sizes (r) and 95% CI are now reported.

**Reviewer comment 8:** Provide baseline characteristics.
New Table 1 with baseline characteristics has been added.

**Reviewer comment 9:** Clarify the role of gender.
It has been clarified now that gender has less influence on the relationship between psychosocial aspects and the decision to accept surgery than age. This text is in conclusion: “In males, heightened awareness of deformity served as a motivation, while in females, lower self-esteem was influential. OF and FE primarily influence decisions, regardless of age or gender.”

## ****Final Statement****

We thank the reviewers for their insightful comments, which significantly improved our manuscript. We believe the revised version addresses all concerns and has been substantially strengthened.

Sincerely,

**Prof.asoc. Jeta Kelmendi**
